# Dynamic Regulation of a Cell Adhesion Protein Complex Including CADM1 by Combinatorial Analysis of FRAP with Exponential Curve-Fitting

**DOI:** 10.1371/journal.pone.0116637

**Published:** 2015-03-17

**Authors:** Mika Sakurai-Yageta, Tomoko Maruyama, Takashi Suzuki, Kazuhisa Ichikawa, Yoshinori Murakami

**Affiliations:** 1 Division of Molecular Pathology, The Institute of Medical Science, The University of Tokyo, 4-6-1, Shirokanedai, Minato-ku, Tokyo, 108-8639, Japan; 2 Division of Mathematical Oncology, The Institute of Medical Science, The University of Tokyo, 4-6-1, Shirokanedai, Minato-ku, Tokyo, 108-8639, Japan; 3 Japan Science and Technology Agency, CREST, 4-5-3, Yonbancho, Chiyoda-ku, Tokyo, 102-8666, Japan; 4 The Division of Mathematical Science, Graduate School of Engineering Science, Osaka University, 1-3, Machikaneyama-cho, Toyonaka, Osaka, 560-8531, Japan; Vanderbilt University Medical Center, UNITED STATES

## Abstract

Protein components of cell adhesion machinery show continuous renewal even in the static state of epithelial cells and participate in the formation and maintenance of normal epithelial architecture and tumor suppression. CADM1 is a tumor suppressor belonging to the immunoglobulin superfamily of cell adhesion molecule and forms a cell adhesion complex with an actin-binding protein, 4.1B, and a scaffold protein, MPP3, in the cytoplasm. Here, we investigate dynamic regulation of the CADM1-4.1B-MPP3 complex in mature cell adhesion by fluorescence recovery after photobleaching (FRAP) analysis. Traditional FRAP analysis were performed for relatively short period of around 10min. Here, thanks to recent advances in the sensitive laser detector systems, we examine FRAP of CADM1 complex for longer period of 60 min and analyze the recovery with exponential curve-fitting to distinguish the fractions with different diffusion constants. This approach reveals that the fluorescence recovery of CADM1 is fitted to a single exponential function with a time constant (τ) of approximately 16 min, whereas 4.1B and MPP3 are fitted to a double exponential function with two τs of approximately 40-60 sec and 16 min. The longer τ is similar to that of CADM1, suggesting that 4.1B and MPP3 have two distinct fractions, one forming a complex with CADM1 and the other present as a free pool. Fluorescence loss in photobleaching analysis supports the presence of a free pool of these proteins near the plasma membrane. Furthermore, double exponential fitting makes it possible to estimate the ratio of 4.1B and MPP3 present as a free pool and as a complex with CADM1 as approximately 3:2 and 3:1, respectively. Our analyses reveal a central role of CADM1 in stabilizing the complex with 4.1B and MPP3 and provide insight in the dynamics of adhesion complex formation.

## Introduction

Cell adhesion machinery, composed of cell adhesion molecules, cytoskeletal proteins, and scaffolding proteins, are spatiotemporally regulated in the maintenance of epithelial structure. This machinery includes tight junctions (TJ), adherens junctions (AJ), and desmosomes, as well as various other protein complexes that participate in cell adhesion in a static manner [1,2]. Maintenance of such cell-cell adhesion in epithelia also protects cells from malignant conversion, including invasion and metastasis [3]. However, FRAP analysis has revealed that the protein components of cell adhesion machinery show continuous renewal at the molecular level with a halftime of recovery (t_1/2_) of less than a few minutes even in the static state of epithelial cells. For example, a membrane protein, occludin, and its cytoplasmic binding protein, ZO-1—representative components of TJ—are dynamically regulated in confluent cells with t_1/2_ of 107s and 98s, respectively, although TJ machinery is expected to be stably responsible for its strong barrier function [4]. Similarly, AJ component proteins, such as E-cadherin and its cytoplasmic binding partners, β-catenin, α-catenin, and actin, are continuously exchanged with each other in the static state of cell-cell adhesion [5]. Thus, cell adhesion machinery appears to be remodeled quite elaborately in the formation and maintenance of the epithelial architecture. However, dynamic regulation of other cell adhesion molecule complex, as well as its individual components, is not yet fully understood.

We have previously identified Cell adhesion molecule 1 (CADM1) as a tumor suppressor in non-small cell lung cancer (Gene ID: 23705) [6]. CADM1 is an immunoglobulin superfamily cell adhesion molecule (IgCAM) expressed in most of the epithelial and neuronal synapses [7], and is also called *TSLC1* [6], *Necl-2* [8], and *synCAM1* [9]. In polarized epithelial cells, CADM1 is not localized in TJ or AJ but expressed diffusely in the lateral membrane as homodimers, *trans*-interacts with CADM1 from adjacent cells, and participates in cell adhesion [10]. Expression of CADM1 in single-cell suspended MDCK cells causes cell aggregation in Ca^2+^ or Mg^2+^ independent manner [10]. The cytoplasmic domain of CADM1 contains two protein interaction motifs, a protein 4-binding motif (4.1-BM) and a class II PDZ-binding motif (PDZ-BM). We have previously demonstrated that, through 4.1-BM, CADM1 interacts with protein 4.1B/DAL-1 [11], which further binds to spectrin, an actin-binding protein [12], and localizes CADM1 to the cell-cell contact sites with actin [11]. CADM1 is also associated with a member of PDZ domain-containing membrane-associated guanylate kinases (MAGuKs), including MPP1–3, CASK and Pals2 [8,9,13,14] through a class-II PDZ-BM. In HEK293 or Caco-2 cells, depletion of CADM1 by siRNA led to loss of actin bundle maturation and epithelial morphology [14]. On the other hand, MDCK cells expressing CADM1 show spread morphology like that in an initial phase of cell adhesion when cultured on the immobilized extracellular fragments of CADM1 through *trans*-homophilic interaction. This spread morphology is inhibited by cytochalasin B, indicating that CADM1 transmits the cell attachment signals to the morphological changes through actin-reorganization [15]. In addition, expression of CADM1 in MDCK cells suppressed the epithelial to mesenchymal transition (EMT) triggered by HGF treatment, whereas mutant CADM1 lacking its cytoplasmic fragment lost suppressor activity of EMT by HGF [16]. Taken together, these findings suggest that CADM1–4.1B-MPP3 protein complex is involved in the formation and maintenance of epithelial cell structure.

By contrast, CADM1 expression is often lost by promoter methylation in various invasive cancers, including non-small cell lung cancer, breast cancer and pancreatic cancer [7]. 4.1B expression is also lost in lung, breast and renal cancers [17–19]. Notably, the expression of either CADM1, 4.1B or MPP3 is lost in 11 of 12 lung tumor cell lines, suggesting that these proteins provide an important cascade for tumor suppressor. Correspondingly, restoration of CADM1 expression in human lung cancer cell line, A549, suppressed their tumorigenicity in nude mice, whereas mutant CADM1 lacking its cytoplasmic domain, 4.1-BM, or PDZ-BM, lost its suppressor activity in subcutaneous tumor formation in nude mice [20]. These findings suggest that the ternary complex of CADM1–4.1B-MPP3 is also crucial for tumor suppression.

In physiological situations, CADM1 appears to be dynamically regulated on the plasma membrane since rapid accumulation of CADM1 was observed in the early stage of cell-cell contact, even though the amount of CADM1 protein was not changed before or after the formation of cell-cell contact [10]. Expression of an appropriate amount of CADM1 is considered to be important for epithelial structure/function, because formation of mature adhesion was abrogated by the depletion of CADM1 using its siRNA [14], and because wound healing in the skin was impaired in the *Cadm1*-deficient mice as well as in the *Cadm1* transgenic mice [21]. Furthermore, we previously found that CADM1-binding proteins 4.1B and MPP2 lost their juxtamembrane localization and dispersed in the cytoplasm of cells when CADM1 was depleted, even though the amounts of 4.1B or MPP2 proteins were not affected [14]. These findings suggest that appropriate amount of CADM1 expression regulates subcellular localization and the stability of its binding proteins at cell-cell contact sites.

Here, we investigated the dynamic regulation of the CADM1 complex in epithelial cells, MDCK. Although endogenous CADM1 is scarcely detected in MDCK cells, exogenous expression of CADM1 in MDCK cells leads to cell aggregation [10], suppresses experimental EMT triggered by HGF [16], and induces spreading morphology caused by actin reorganization and *trans*-homophilic interaction with immobilized CADM1 [15], indicating that CADM1 plays physiological roles in the formation and maintenance of epithelial structure in MDCK cells. FRAP analysis for 60 min and subsequentexponential curve-fitting demonstrated that CADM1 was present as a relatively stable fraction independent of the complex formation, whereas its binding proteins, 4.1B and MPP3, were present as a stable and a unstable state, which might correspond to the fractions present as a CADM1 complex and a free pool, respectively.

## Results

### Dynamics of CAMs in Confluent Cells Analyzed by t_1/2_ and Mf

**Fig 1:**
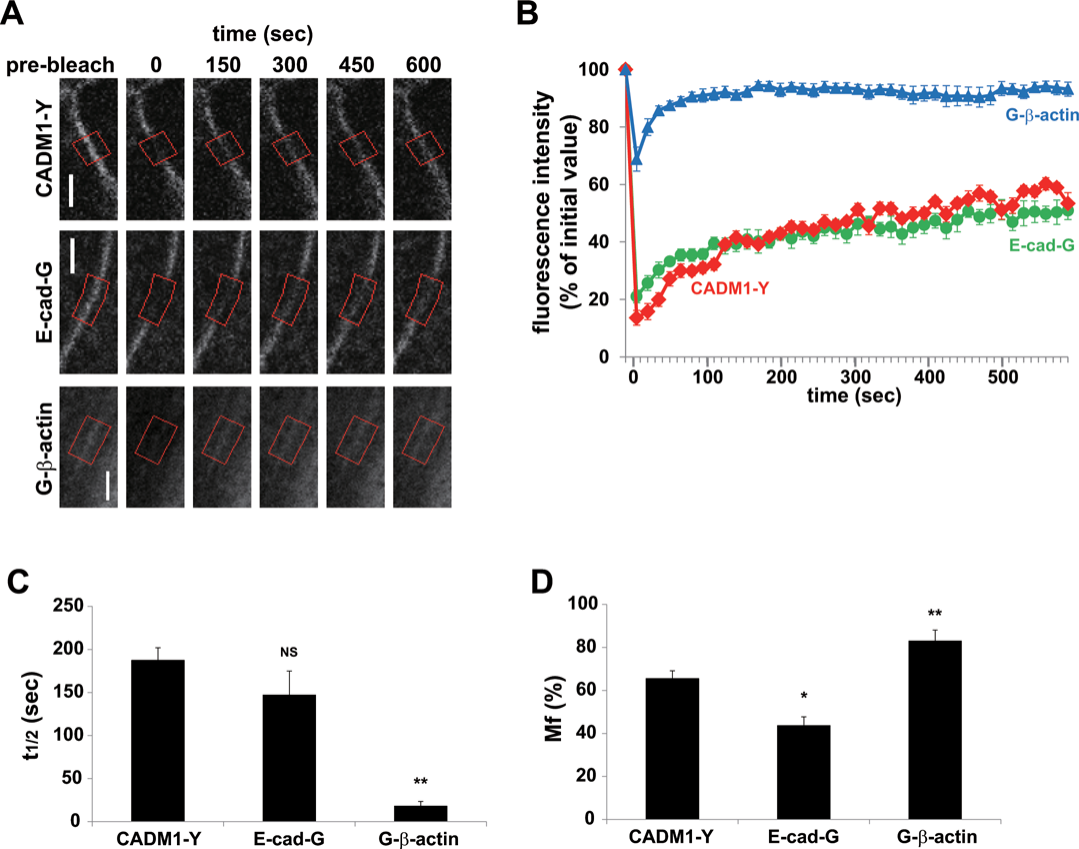
Conventional FRAP analysis of CAMs expressed in confluent MDCK cells. MDCK cells expressing CADM1-Y, E-cadherin-G, or G-β-actin were analyzed using FRAP for 600 sec (short, **A**–**D**) after photobleaching. (**A**) Representative images before and at the time points indicated after photobleaching are shown. ROIs for photobleaching are indicated by red boxes. Bars, 5 μm. (**B**) Fluorescence recovery curve of FRAP analysis. (**C** and **D**) Halftime of recovery (t_1/2_, **C**) and mobile fraction (Mf, **D**). Data are mean ± SEM. Statistical differences in t_1/2_ and Mf in FRAP analysis for 10min were determined by Student’s t-test. *, p< 0.05; **, p< 0.01; NS, no significant difference. (**B**–**D**) n = 10, 5, and 8 for CADM1-Y, E-cadherin-G, and G-β-actin, respectively.

We initially analyzed the dynamics of two cell adhesion molecules, CADM1 and E-cadherin, as well as β-actin, in confluent MDCK cells expressing these molecules tagged with GFP (G) or YFP (Y) (S1A-S1C Fig.). Only a trace amount of CADM1 was endogenously expressed in the parental MDCK cells. We found that CADM1-Y, like endogenous CADM1, was expressed in cell-cell attachment sites on the membrane by con-focal microscopy and that CADM1-Y gave a value similar to that of CADM1 by measuring the trans-epithelial resistance (TER) of epithelial sheets formed by CADM1-Y transfected cells (S1D Fig.). These cells were continuously cultured for additional 4 days after the cells reached confluence, and the dynamics of fluorescent proteins in the cell-cell adhesion sites were examined using FRAP analysis. In this analysis, the fluorescence of each protein was photobleached in part of a cell-cell contact site, and its recovery was monitored for 10 min consistent with previous studies by other investigators (Fig. 1A) [4,22]. Using conventional quantitative analysis, G-β-actin was rapidly exchanged with a t_1/2_ of 18.4 ± 5.5 sec and a Mf of 83.0 ± 5.0% (Fig. 1B-1D, S1 Table) similar to those previously reported [5]. In this condition, the CADM1-YFP (CADM1-Y) and E-cadherin-GFP (E-cadherin-G) showed Mfs of 65.5 ± 3.6% and 43.6 ± 4.0%, respectively. The rates of exchange of CADM1-Y and E-cadherin-G are shown with t_1/2_ of 187 ± 14 sec and 147 ± 28 sec, respectively (S1 Table). The dynamics of E-cadherin were similar to those previously reported, showing an Mf range of 23–65% and a t_1/2_ of 0.5–10 min [5,23].

**Fig 2:**
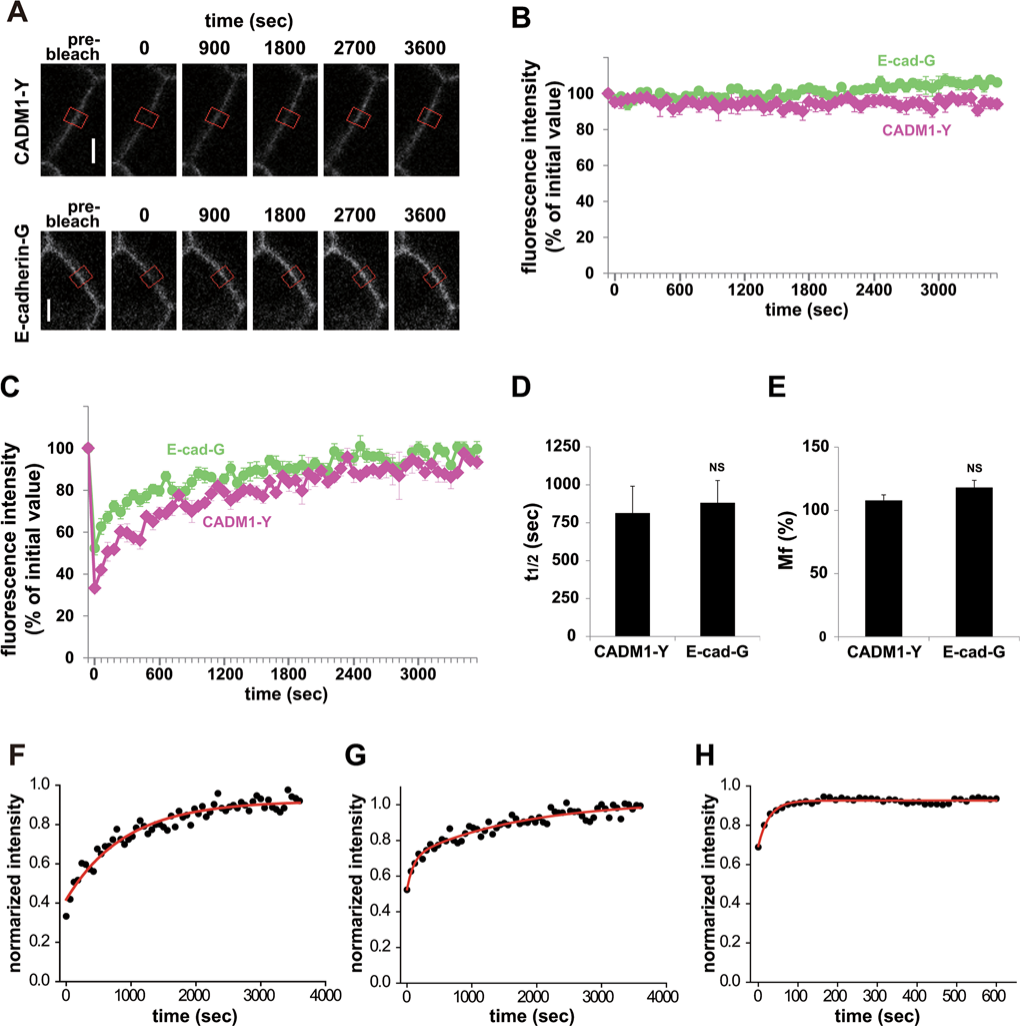
Dynamics of CAMs, CADM1, and E-cadherin in confluent MDCK cells. MDCK cells expressing CADM1-Y or E-cadherin-G were analyzed using FRAP for 3,600 sec after photobleaching. (**A**) Representative images before and at the time points indicated after photobleaching are shown. ROIs for photobleaching are indicated by red boxes. Bars, 5 μm. (**B**) Fluorescence intensity in the control ROI without photobleaching. (**C**) Fluorescence recovery curve of FRAP analysis. (**D** and **E**) Halftime of recovery (t_1/2_, **D**) and mobile fraction (Mf, **E**). Data are mean ± SEM. Statistical differences in t_1/2_ and Mf in FRAP analysis for 60min were determined by Student’s t-test. NS, no significant difference. (**F**–**H**) Single or double exponential curve fitting of fluorescence intensities of CADM1-Y (**F**, n = 5), E-cadherin-G (**G**, n = 9), and G-β-actin (**H**, n = 8). Exponential fitting for FRAP signals was performed as described previously [24]. To determine the time constant of fluorescence recovery at the cell-cell contact sites, the experimental data were plotted in a semi-logarithmic scale, and regression analysis was performed using the following equation:*y* = *y*_0_ + *A*_1_ ·exp(—*x*/*τ*_1_) + *A*_2_ exp(—*x*/*τ*_2_)

One concern with these experiments is that the recovery curves of CADM1-Y and E-cadherin-G did not reach plateaus within 10 min of analysis. Thus, we challenged to analyze the fluorescence recovery for a longer time period, 60 min (Fig. 2A). We confirmed that the fluorescence loss was not seen in the control ROI without analysis of long-term photobleaching for 60 min (Fig. 2B). Regression analysis showed that t_1/2_s of the CADM1-Y and E-cadherin-G were 811.7 ± 179.3 sec and 879.5 ± 149.4 sec, respectively, with Mfs of 107.7 ± 4.5% and 117.8 ± 6.0%, respectively, when calculated using the equations for regression analysis described in Materials and Methods (Fig. 2C, 2D and 2E, Table 1) [4, 25]. These t_1/2_ and Mfs are much larger than those in both CADM1-Y and E-cadherin-G obtained by 10-min FRAP analysis. These results also show that FRAP analysis can be practically performed for longer time period of up to 60 min.

**Table 1:** The time constant (τ) of E-cadherin, CADM1, 4.1B and MPP3 analyzed by long-time.

	Single transfectant			Double tranfectant		Double tranfectant	
	CADM1-Y	E-cadherin-G	β-actin-G	CADM1-Y	G-4.1B	CADM1-Y	G-MPP3
	(single^a^)	(double^b^)	(single^a^)	(single^a^)	(double^b^)	(single^a^)	(double^b^)
R^2^	0.936	0.921	0.935	0.927	0.878	0.920	0.858
y0	0.921	1.021	0.926	80.58	89.06	78.78	94.94
	± 0.011	± 0.030	±0.002	± 1.36	± 1.07	± 1.22	± 1.00
A1	0.503	0.177	0.232	45.15	26.46	48.65	31.25
	± 0.017	± 0.036	± 0.011	± 1.63	± 3.73	± 1.88	± 3.16
τ1	907.5s	97.55s	27.68s	1084s	62.65s	917.3s	41.21s
	± 80.00s	± 41.97s	± 2.43s	± 113.4s	± 19.60s	± 91.77s	± 11.27s
A2	-	0.318	-	-	17.72	-	11.51
	-	± 0.020	-	-	± 2.32	-	± 1.69
τ2	-	1689s	-	-	963.0s	-	1010s
	-	± 462.3s	-	-	± 275.1s	-	± 372.7

^a^Single exponential-fitting;

^b^Double exponential-fitting.

### Analysis of dynamic regulation of CAMs by exponential curve fitting

Next, we analyzed the recovery of FRAP signals using another approach by fitting them with an exponential function for 60 min. If the recovery process is random, such as diffusion, the recovery of FRAP signals can be fitted by an exponential function [24], and if the recovery is composed of two independent random processes, the recovery of FRAP signals would be fitted by an exponential function with two different time constants and amplitudes. The recovery curve of CADM1-Y was fitted by a single exponential function with a τ of 907.5 ± 80.0 sec (R^2^ = 0.936), suggesting that CADM1-Y was stably localized at the cell-cell contact sites for an average of about 15 min (Fig. 2F and Table 1). In contrast, the recovery of E-cadherin-G was fitted by a double exponential function with a τ of 97.5 ± 41.9 sec (R^2^ = 0.921) and 1,689 ± 462 sec, suggesting that E-cadherin-G at the cell adhesion sites was present in the mixture of an unstable fraction exchanged within a few minutes and a stable fraction, of which τ is larger than that of CADM1-Y (Fig. 2G and Table 1). On the other hand, β-actin showed rapid exchange in this analysis with a single τ of 27.7 ± 2.4 sec (R^2^ = 0.935), which is similar to that obtained in short-term analysis (Fig. 2H and Table 1).

### Two Different Time Constants of CADM1 Binding Proteins, 4.1B and MPP3

**Fig 3:**
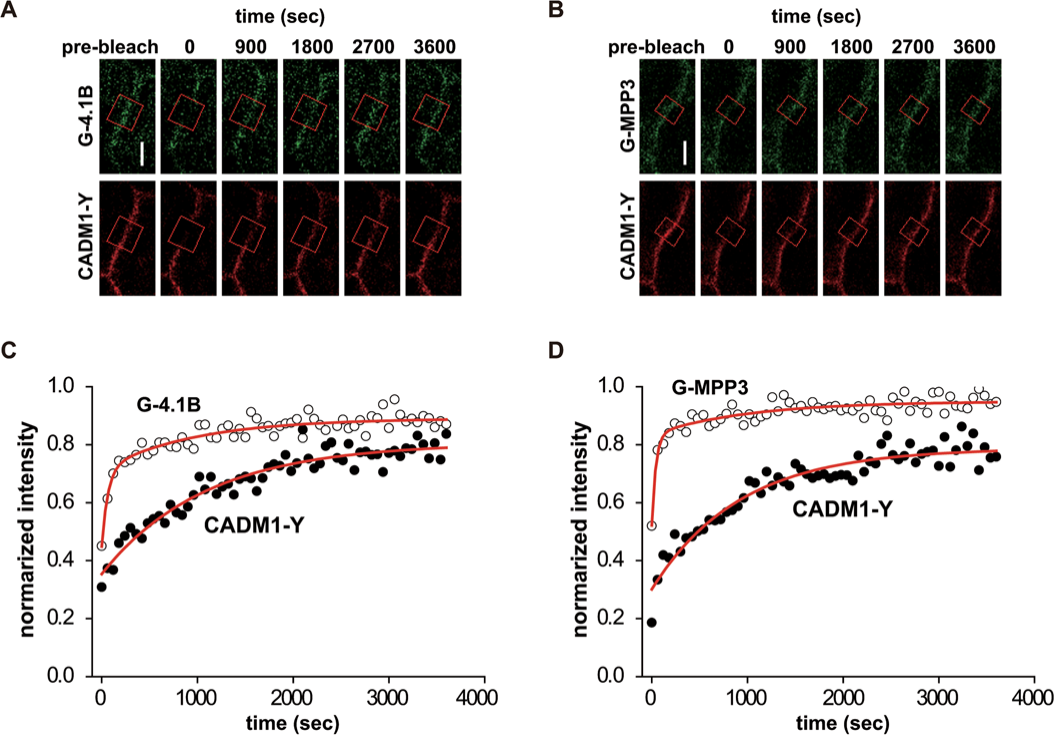
Dynamics of CADM1 and its binding proteins, 4.1B and MPP3, at cell-cell contact sites. MDCK cells expressing CADM1-Y and G-4.1B (**A** and **C**) or G-MPP3 (**B** and **D**) were analyzed using FRAP until 3,600 sec after photobleaching. (**A** and **B**) Representative images before and at the time points indicated after photobleaching are shown. ROIs for photobleaching are indicated by red boxes. Bars, 5 μm. (**C** and **D**) Single or double exponential curve fitting of fluorescence intensities of cells expressing CADM1-Y/G-4.1B (**C**, n = 7) and CADM1-Y/G-MPP3 (**D**, n = 8) as indicated in Table 1.

**Fig 4:**
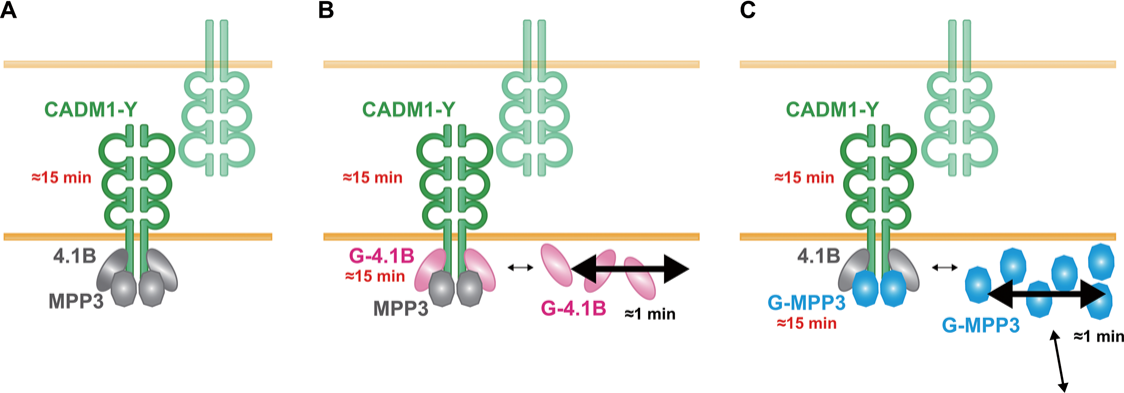
A schematic representation of the dynamics of the CADM1 complex. In confluent MDCK cells, CADM1-Y forms *cis*-dimers on plasma membranes and *trans*-interacts with CADM1-Y expression in the adjacent cell. (**A**) In the single transfectant, CADM1-Y interacted with endogenous proteins 4.1s and MPPs and stably localized at the cell-cell contact sites with a time constant of approximately 16 min. In double transfectants of CADM1-Y and G-4.1B (**B**) or G-MPP3 (**C**), CADM1-Y was still stably localized; in contrast, G-4.1B or G-MPP3 was present as a free pool or as a complex with CADM1-Y with short- or long-time constants, respectively. The time constant of each protein is indicated.

Then, we examined the dynamics of components of the CADM1 complex. For this purpose, MDCK cells expressing CADM1-Y were further transfected with G-4.1B or G-MPP3 (S1A-B Fig.). Relative expression of CADM1-Y:G-4.1B is estimated to be 8:1, while that of CADM1-Y:G-MPP3 is 1:2 by immunoblot analysis using anti-GFP antibody (S1C Fig.). In addition, relative expression of exogenous G-4.1B and G-MPP3 to endogenous 4.1B and MPP3 are 5:1 and 7:1, respectively (S1B Fig.). We performed FRAP analysis for 60 min using these cells and monitored the fluorescence recovery of CADM1-Y and G-4.1B or G-MPP3 at the same time (Fig. 3A and 3B). In the double transfectant of CADM1-Y and G-4.1B, the recovery curve of CADM1-Y was fitted by a single exponential function with a τ of 1,084 ± 113.4 sec (R^2^ = 0.927) similarly to its single transfectant (Fig. 3C and Table 1), indicating that the stability of CADM1 is not dependent on its complex formation with 4.1B. In contrast, the recovery curve of G-4.1B was fitted by a double exponential function with a short and long τ of 62.6 ± 19.6 sec and 963.0 ± 275.1 sec (R^2^ = 0.878), respectively. The long τ of G-4.1B was similar to the τ of CADM1-Y, suggesting that G-4.1B with a long τ would be present as a complex with CADM1-Y, while G-4.1B with a short τ would be present as a free pool of 4.1B. Similarly, CADM1-Y in the double transfectants with G-MPP3 was fitted by single exponential function with a long τ of 917.3 ± 91.77 sec, whereas G-MPP3 in the same transfectants was fitted by a double exponential function with a short τ of 41.2 ± 11.3 sec and a long τ of 1,010 ± 372.7 sec (R^2^ = 0.858), where the longer τ is similar to that of CADM1-Y (Fig. 3D and Table 1). These findings suggest that G-4.1B and G-MPP3 would be present both as a free pool and as a complex with CADM1. Then, we tried to estimate the fraction of molecules forming complexes with CADM1 by calculating the ratio of amplitudes of these two time constants. Based on comparing *A*_*1*_ with *A*_*2*_ in our experiments, the ratio of G-4.1B and G-MPP3 present as a free pool and as a complex with CADM1-Y was shown to be 26.5:17.7 (approximately 3:2) and 31.2:11.5 (approximately 3:1), respectively (Fig. 4 and Table 1).

### The Rapid Movement of 4.1B and MPP3 Occurred within the Contact Sites

**Fig 5:**
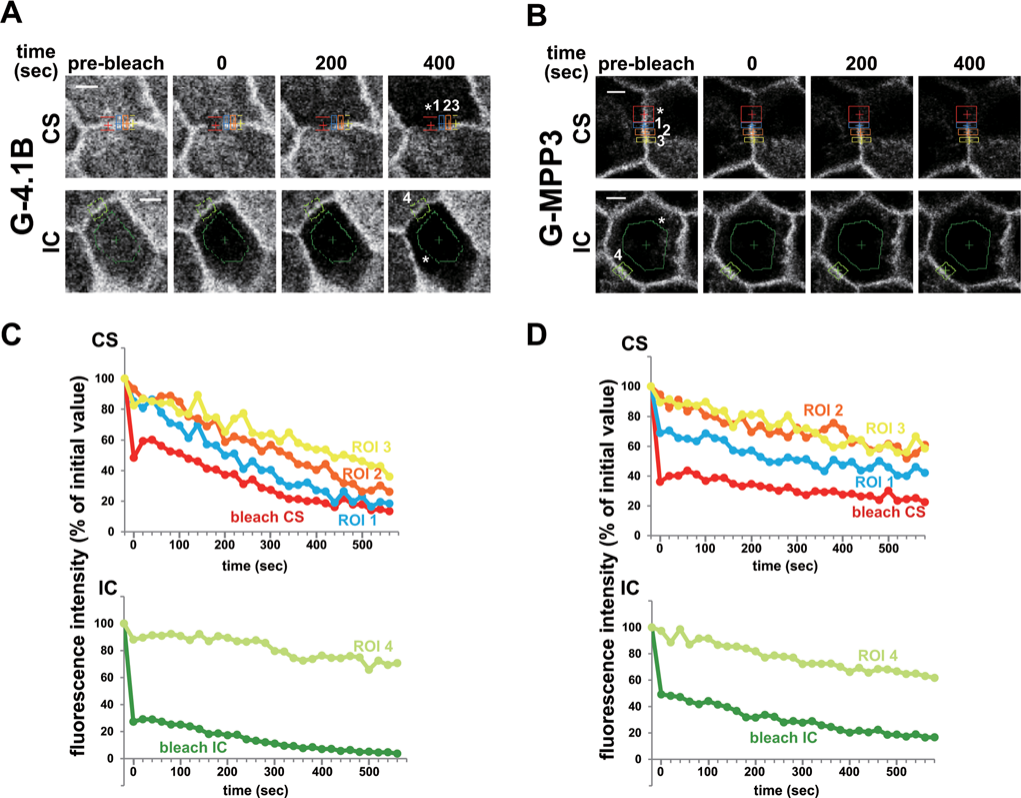
Exchange of CAMs within cell-cell contact regions analyzed using FLIP assay. MDCK cells expressed with CADM1-Y and G-4.1B (**A** and **C**) or G-MPP3 (**B** and **D**) were continuously photobleached at the cell-cell contact site (CS, upper) or intracellular region (IC, lower) indicated by asterisk. (**A** and **B**) Representative images of CS- or IC-bleached cells before and at the time points indicated after photobleaching. Intensity of fluorescent protein was monitored at ROIs (regions of interest) 1–3 in CS-bleached cells and ROI 4 in IC-bleached cells for 600 sec. (**C** and **D**) Quantitative analysis of the fluorescence intensities in the cell-cell contact sites adjacent to the photobleached regions shown in **A** and **B** as ROI 1–4. Quantification was performed in at least three independent experiments, and a representative graph was shown.

Finally, we examined whether the rapid exchange of G-4.1B and G-MPP3 that was implied by a short time constant occurred within the cell-cell contact sites or in the intracellular region by using fluorescence loss in photobleaching (FLIP) analysis. MDCK cells expressing G-4.1B or G-MPP3 were continuously bleached in a small region of the cell-cell contact sites (CS) or the intracellular region (IC), and the fluorescence intensities of adjacent cell-cell contact regions (ROI 1–4) were monitored for 10 min (Fig. 5A and 5B). We failed to continue FLIP analysis for a longer time because the cells were severely damaged by continuous bleaching. The fluorescence intensity of G-4.1B on adjacent cell-cell contact sites was not so much affected by photobleaching in the IC (Fig. 5C). In contrast, bleaching at the CS drastically reduced the fluorescence intensity of G-4.1B within the same contact site according to the distance from the bleached region; G-4.1B in ROI 1 decreased more drastically as compared with that in ROI 3. On the other hand, G-MPP3 localized at the cell-cell contact site was significantly reduced by CS bleaching, while G-MPP3 was moderately affected by IC bleaching (Fig. 5D). These results suggest that the fraction of G-4.1B with a short τ of 1 min was exchanged mainly within the juxtamembrane region, while that of G-MPP3 was replaced not only by G-MPP3 in the juxtamembrane area, but also in the intracellular region.

## Discussion

Dynamic regulation of cellular protein complex is important for understanding their physiological and pathological significance in various biological phenomena, including cell-cell adhesion. For investigating protein dynamics, FRAP and FLIP analyses are most potent and widely used cell biological approaches. A number of previous studies have conventionally used non-linear regression analysis with t_1/2_ and Mf for the quantification of FRAP data, in which t_1/2_ has been reported to be related to the diffusion coefficient [25]. We firstly examined the data by non-linear regression analysis and confirmed that the present experiment worked well because Mf and t_1/2_ obtained in this study were equivalent to those reported previously. Then, we analyzed the data by another approach using an exponential fitting model because this tool can analyze the dynamics of a system composed of multiple random processes, like reaction-diffusion systems. If the signal is better fitted by two exponents, it is strongly suggested that the process is composed of two independent random processes.

Another particular feature of this study is that we performed FRAP analysis for 60 min, which is much longer than 10 min in most reported cases. The cytotoxic effect on the cells caused by long exposure to a high-energy laser beam was a major obstacle that caused us to restrict the observation time to less than 10 min. However, thanks to recent advances in the sensitivity of laser detector systems, long-term FRAP analysis using a low-energy laser beam for up to 60 min becomes possible [26].

In this study, we performed FRAP analysis for 60 min and analyzed the results by exponential curve-fitting. Exponential fitting analyses using the time constants of each protein provides two critical biological findings. First, the dynamics of CADM1 with a single fraction is distinct from that of E-cadherin with two fractions, indicating that CADM1 stability is not dependent on the complex formation with 4.1B or MPP3. *Trans*-homophilic interaction of CADM1 from adjacent cells could be the most important determinant of CADM1 stability in confluent cells as examined in this study. However, considering that E-cadherin is also examined in confluence with *trans*-homophilic interaction, a single random process would be a unique feature of CADM1 in its dynamics. In the case of E-cadherin, a dynamic process mediated by endocytosis [27,28] and another endocytosis-independent process, which would be mediated by lateral diffusion, have been suggested [29,30].

In this connection, it should be noted that the long time experiment might not provide sufficient data points for interpretation of the initial phase of less than 10 min. In fact, we have found that the exponential fitting analysis of a protein in a computational model with τ of 1.00 s is poorly fitted to inappropriate τ of 2.71 s when we analyze only 11 points that are extracted in the initial phase (1 s) from total 51 points for 5 s (τ = 0.825 s), whereas it is well fit to τ of 1.14 s when we analyze 101 points in the initial phase (1 s) from total 501 points for 5 s (τ = 1.05 s) (S2 Fig.). Therefore, careful interpretation of the dynamics of a molecule in the initial phase would be necessary in FRAP analysis of a long time period.

The other important finding in this study is that 4.1B and MPP3 show two time constants of approximately 40–60 sec and 16 min, where the larger ones are the same as that of CADM1 of approximately 16 min. This indicates that 4.1B and MPP3 have two distinct fractions in the dynamics, one forming a complex with CADM1 and the other present as a free pool without forming a complex (Fig. 4). In other words, our study demonstrates that CADM1 is a predominant protein that effectively recruits 4.1B and MPP3 to the cell membrane, indicating the importance of the membrane protein CADM1 in its complex formation with 4.1B and MPP3. It should be noted, however, that the different expression levels of 4.1B and MPP3 relative to that of CADM1 may affect the dynamics of these CADM1-interacting molecules. In this connection, however, Foote HP reported that the dynamics of a tight junction protein ZO-1 was not essentially changed between the cells overexpressing ZO-1 by plasmid transfection and those expressing a physiological ZO-1 level by knock-in system. Additional studies on different cell clones with different expression levels of 4.1B and MPP3 would be helpful to confirm the dynamics of CADM-1 and its binding proteins.

It is also noteworthy that mutant E-cadherin lacking catenin binding site still formed a cluster at the cell-cell contact site [30], suggesting that the interaction of cell adhesion molecules with its cytoplasmic binding proteins was not essential for its stable localization at cell-cell attachment site. With the results from FLIP analysis, we propose that considerable portions of 4.1B and MPP3 are localized stably along the membrane by binding with CADM1, whereas the remaining 4.1B and MPP3 are present as a free pool of rapid exchange. Free 4.1B would pool under the plasma membrane, probably through interacting with the actin cytoskeleton along the cortex in confluent cells. A portion of free MPP3 might also pool in the juxtamembrane area, possibly through its direct interaction with protein 4.1s through the HOOK domain [31], whereas another portion of the free MPP3 could be supplied from the intracellular region, possibly through protein trafficking as shown in other members of the MAGuK subfamily such as ZO-1 and SAP97/hDlg [4,32]. The free pools under the membrane would be beneficial for timely and coordinated remodeling of cell adhesion machinery of CADM1–4.1B-MPP3.

In this study, we could also show that the ratio of G-4.1B and that of G-MPP3 present as a free pool and as a complex with CADM1-Y is approximately 3:2 and 3:1, respectively (Fig. 4 and Table 1). Considering that the fraction with slow-turnover is higher in 4.1B (40%) than in MPP3 (25%), possible difference in the sensitivity of 4.1B and MPP3 to IC bleaching may simply reflect lower turnover of 4.1B in CS rather than a different distribution. Moreover, these ratios could be also affected by the expression level of CADM1-Y and G-4.1B or G-MPP3. In fact, if we take it into account that the expression level of G-4.1B is much lower than that of CADM1-Y in our experiment, presence of around 60% of G-4.1B as a free pool suggests that CADM1-Y might associate with some other proteins, such as 4.1N [33]. On the other hand, higher expression of MPP3-G relative to that of CADM1 in the doubly transfected MDCK cells could be one of the mechanisms to explain the high proportion of free MPP3-G.

To the best of our knowledge, this is the first demonstration of the dynamic analysis of multiple protein complexes of cell adhesion machinery using exponential curve-fitting of FRAP in combination with FLIP analysis. We recognize that this is one of the approaches to understand the dynamics of protein complex on the cell membrane and that some other approaches using non-linear regression model [34] or reaction diffusion kinetics model [35] would be also applicable and helpful to understand the whole view of the dynamic features of membrane proteins. Although we interpreted the number of τ as the number of different pools in which a protein may exist in this study, a protein with a single pool may sometimes show two τ in a model of reaction diffusion kinetics, because fast diffusion kinetics followed by slow binding kinetics has been reported to have two recovery components in a FRAP curve, although time scale of diffusion is much faster than that of binding kinetics [34,35]. Moreover, anomalous diffusion FRAP model could show a similar multi-component FRAP curve [36]. On the other hand, present study demonstrates that 4.1B and MPP3 have a fast pool and a slow pool, each appears to be corresponding to a free pool and a bound pool with CADM1 by analyzing the data by exponential curve fitting. This mathematical approach would shed new light on the mechanical understanding of the dynamics of protein complexes in the cells.

## Materials and Methods

### Cell culture and transfection

MDCK cells were cultured as described previously [10]. Cells were transfected with the relevant plasmids using Lipofectamine LTX (Invitrogen) and selected with 200 μg/ml of hygromycin (Invitrogen) or 500 μg/ml of Geneticin (Invitrogen). Stable cell clones of MDCK were cultured for about 5 days after reaching confluence on ibidi-treated plastic bottom dishes (ibidi).

### Expression vectors

CADM1 expression vector has been described previously [6]. Human E-cadherin, and β-actin cDNAs were cloned using RT-PCR from human lung poly A^+^ RNA (Clontech), while human 4.1B and MPP3 cDNAs were cloned as described previously [11,13]. E-cadherin cDNA was inserted into pEGFP-N3, and β-actin, 4.1B, and MPP3 cDNAs were cloned into pEGFP-C3 (Clontech) to obtain E-cadherin-GFP (E-cad-G), GFP-β-actin (G-β-actin), GFP-4.1B (G-4.1B), and GFP-MPP3 (G-MPP3), respectively. To generate the expression vector of CADM1 tagged with YFP in the region between the extracellular and transmembrane fragments (CADM1-Y), CADM1 (aa 1–373 and 374–443) and YFP cDNA were amplified separately using PCR to generate overlapped fragments and fuse them using secondary PCR; the fragments obtained were then cloned into pcDNA3.1/Hygro(+) (Invitrogen). The structure of CADM1-Y is identical to that reported previously [37] and its localization and cell adhesion activity was confirmed by confocal microscopy and TER, respectively. All constructs were sequenced to confirm the inserts.

### Fluorescence recovery after photobleaching (FRAP) analysis

A FRAP experiment was performed using Zeiss lsm 710 confocal microscope system with a 63x oil-immersion objective and a 3.2 AU pinhole corresponding to a 2.4 μm section. Spectral images within the 495–601 nm range were captured every 9.6 nm using 488 nm laser excitation with 0.2–0.3% laser power and unmixed into GFP and YFP images online. A region of interest (ROI) that covered one third of the cell-cell contact site was bleached using the maximum laser power (488 nm) with 15 iterations. The fluorescence intensity immediately after bleaching was set to 0. Images were taken every 15 sec for 10 min or every 60 sec for 60 min to monitor fluorescence recovery. Fluorescence of the ROIs was adjusted by background subtraction at each time point. No significant loss of fluorescence in cell-cell contact sites without bleaching was confirmed throughout the experiments. The t_1/2_ and Mf were analyzed with nonlinear regression software, Sigma Plot 11, using the following equations as reported previously [4,25].

$$I(t)=\frac{I_{0}+I_{\max}\times t/t_{1/2}}{1+t/t_{1/2}}.$$

Mf was determined as:

$$Mf=\frac{I_{\max}-I_{0}}{1-I_{0}}$$

Exponential fitting for FRAP signals was performed as described previously [24]. To determine the time constant (τ) of fluorescence recovery at the cell-cell contact sites, the regression analysis was performed using the following equation in OriginPro 8.5:

y=y0+A1⋅exp(−x/τ1)+A2⋅exp(−x/τ2)

### Fluorescence loss in photobleaching (FLIP) analysis

FLIP analysis was performed using the Nikon A1R microscope system with a 63x oil-immersion objective and a 1.6 AU pinhole corresponding to a 1.2 μm section. ROIs on the cell-cell contact site (CS) or intracellular (IC) region were bleached every 12–20 sec using 15% and 30% of maximum laser power, respectively, and images were taken just after bleaching for 12 min. Fluorescence of the ROIs in continuous bleaching and those in adjacent cell-cell contact sites was quantified and adjusted similarly to the FRAP analysis.

### Exponential fitting analysis of a computed model

A computed model of a protein dynamics was constructed by the data set given by

$$\;\text{y}=\left(1-exp^{-t/\tau }\right)\left(1+2A\gamma \right),$$

where *A* (= 0.2) is the amplitude of the white nose(γ). γ is-0.5 ~ +0.5 and τ is 1 s. We examined two cases at the total numbers of data point with 501 (S2A Fig.) and 51 (S2B Fig.), from which 101 and 11 points (S2C-D Fig., respectively) in the initial phase of 20% are extracted, respectively, and used for the analysis of exponential curve fitting.

### Statistical analysis

Statistical differences in t_1/2_ and Mf in FRAP analysis for 10min were determined by Student’s t-test.

We used OriginPro 8.5.0J SR1 from OriginLab Coorporation for the exponential curve fitting. This software fits given data points with various functions including single and double exponential functions, and gives us time constant(s), amplitude(s) together with R^2^.

## Supporting Information

S1 Table Mf and T_1/2_ estimated by FRAP analysis for 10 min (short time) or 60 min (long time). (DOCX)

S1 Fig MDCK cells expressing fluorescent proteins used for analysis. (**A**) A scheme of CADM1-Y, E-cadherin-G, G-4.1B, and G-MPP3. (**B**) MDCK cells stably expressing fluorescence protein shown in **A** were analyzed by immunoblotting, using specific antibodies against CADM1 (c-18, upper left), E-cadherin (Clone 36, BD Biosciences, upper right), 4.1B (N, lower left), or MPP3 (L27, lower right) as described previously [14]. Black arrows indicate exogenous proteins, while white arrows show endogenous proteins. (**C**) Double transfectants ofCADM1-Y and G-4.1B or G-MPP3 were analyzed by immunoblotting using mouse monoclonal anti-GFP antibodies (Roche Diagnostics). Note that CADM1-Y-expressing clone was further transfected with the expression vector of GFP-fusion protein to obtain CADM1-Y/G-4.1B and CADM1-Y/G-MPP3 clones. The signal around 100 kDa in the lane of CADM1-Y/G-4.1B could be degraded G-4.1B (asterisk). Expression of GAPDH was similarly analyzed as a loading control. (**D**) Transepithelial resistance (TER) of MDCK cells expressing CADMs; cells were cultured on a collagen I-coated Transwell membrane filter with a 3-μm pore (BD) and analyzed with a monitoring TER with cellZscope (CellSeed Inc.) until it reached a plateau at a confluence. Data are mean ± SEM of two independent experiments. TER of MDCK cells were not affected by overexpression of CAMs.
(TIF)

S2 Fig Exponential fitting to data points generated by a theoretical model with nose. Data points are generated by an equation y = (1-*exp* ^-*t*/*τ*^)(1 + 2*Aγ*), assuming a single random process. **A** (= 0.2) is the amplitude of the white nose (γ). γ is-0.5 ~ +0.5 and τ is 1 s. An exponential fitting to the data of 501 points for 5 s is almost perfect (τ = 1.05 s in **A**), while there is a significant error in the fitting to 51 points data (τ = 0.825 s in **B**). Fitting to the initial 1 s data with 101 points, which are extracted from **A** is fairly good (**C**), but fitting to 11 points from **B** is poor, although R^2^ is 0.925 (**D**).
(TIF)
