# Supplementary material for: Dynamic Regulation of a Cell Adhesion Protein Complex Including CADM1 by Combinatorial Analysis of FRAP with Exponential Curve-Fitting
Source: PLoS One. 2015 Mar 17;10(3):e0116637. doi: 10.1371/journal.pone.0116637 (PMC4364555; doi:10.1371/journal.pone.0116637)
Supplement: S1 Table — (DOCX) [file pone.0116637.s001.docx]

**Supplemental Table 1. Mf and T_1/2_ estimated by FRAP analysis for 10 min (short time) or 60 min (long time)**

|  | Mf (%) | T_1/2_ (sec) |
| --- | --- | --- |
| (short time) |  |  |
| CADM1-Y | 65.5 ± 3.6 | 187 ± 14 |
| E-cadherin-G | 43.6 ± 4.0 | 147 ± 28 |
| G-β-actin | 83.0 ± 5.0 | 18.4 ± 5.5 |
| (long time) |  |  |
| CADM1-Y | 107.7 ± 4.5 | 811.7 ± 179.3 |
| E-cadherin-G | 117.8 ± 6.0 | 879.5 ± 149.4 |
