# Supplementary figures and images for: Dynamic Regulation of a Cell Adhesion Protein Complex Including CADM1 by Combinatorial Analysis of FRAP with Exponential Curve-Fitting
Source: PLoS One. 2015 Mar 17;10(3):e0116637. doi: 10.1371/journal.pone.0116637 (PMC4364555; doi:10.1371/journal.pone.0116637)

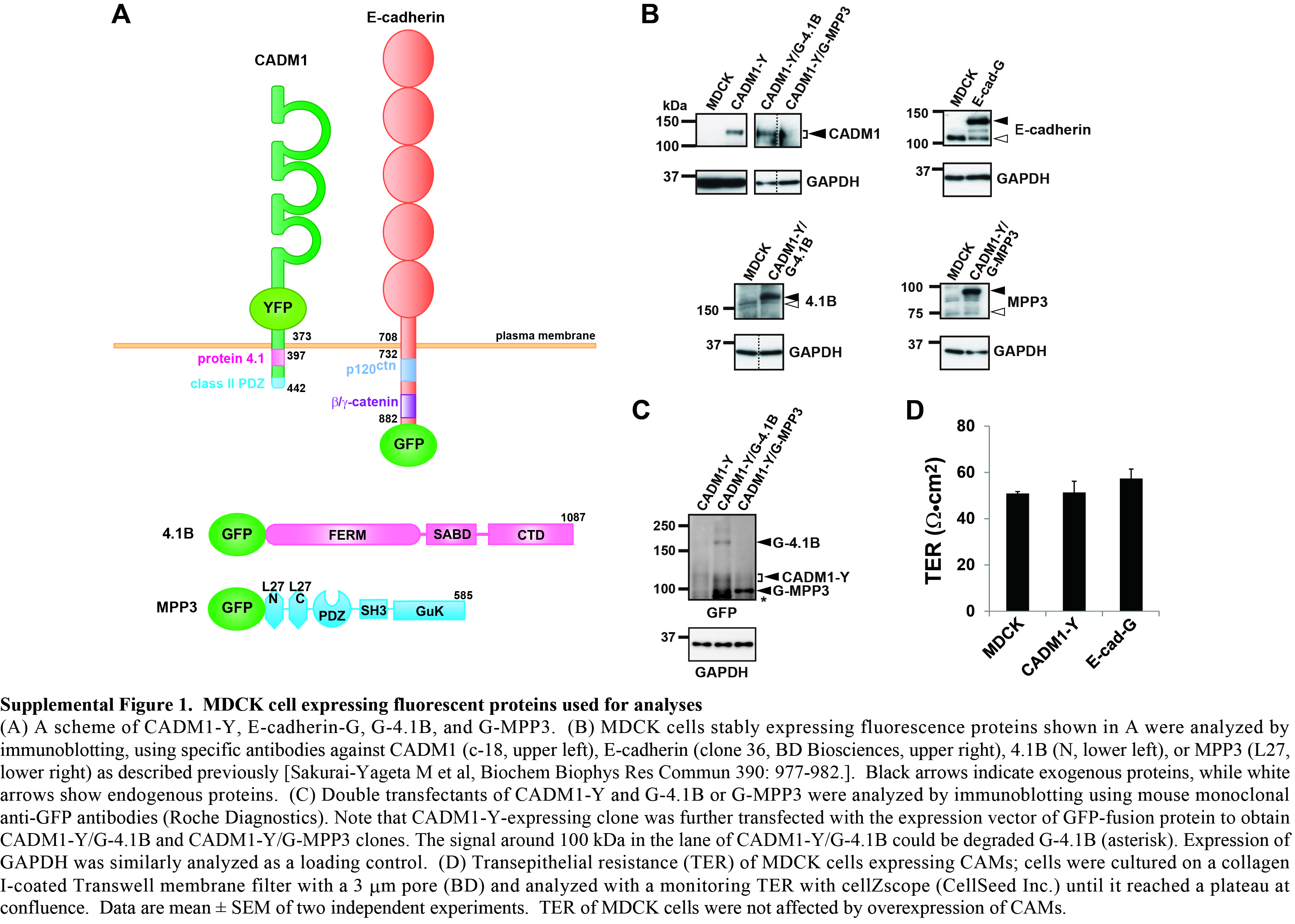

Supplement: S1 Fig — (A) A scheme of CADM1-Y, E-cadherin-G, G-4.1B, and G-MPP3. (B) MDCK cells stably expressing fluorescence protein shown in A were analyzed by immunoblotting, using specific antibodies against CADM1 (c-18, upper left), E-cadherin (Clone 36, BD Biosciences, upper right), 4.1B (N, lower left), or MPP3 (L27, lower right) as described previously [14]. Black arrows indicate exogenous proteins, while white arrows show endogenous proteins. (C) Double transfectants ofCADM1-Y and G-4.1B or G-MPP3 were analyzed by immunoblotting using mouse monoclonal anti-GFP antibodies (Roche Diagnostics). Note that CADM1-Y-expressing clone was further transfected with the expression vector of GFP-fusion protein to obtain CADM1-Y/G-4.1B and CADM1-Y/G-MPP3 clones. The signal around 100 kDa in the lane of CADM1-Y/G-4.1B could be degraded G-4.1B (asterisk). Expression of GAPDH was similarly analyzed as a loading control. (D) Transepithelial resistance (TER) of MDCK cells expressing CADMs; cells were cultured on a collagen I-coated Transwell membrane filter with a 3-μm pore (BD) and analyzed with a monitoring TER with cellZscope (CellSeed Inc.) until it reached a plateau at a confluence. Data are mean ± SEM of two independent experiments. TER of MDCK cells were not affected by overexpression of CAMs. (TIF) [file pone.0116637.s002.tif]

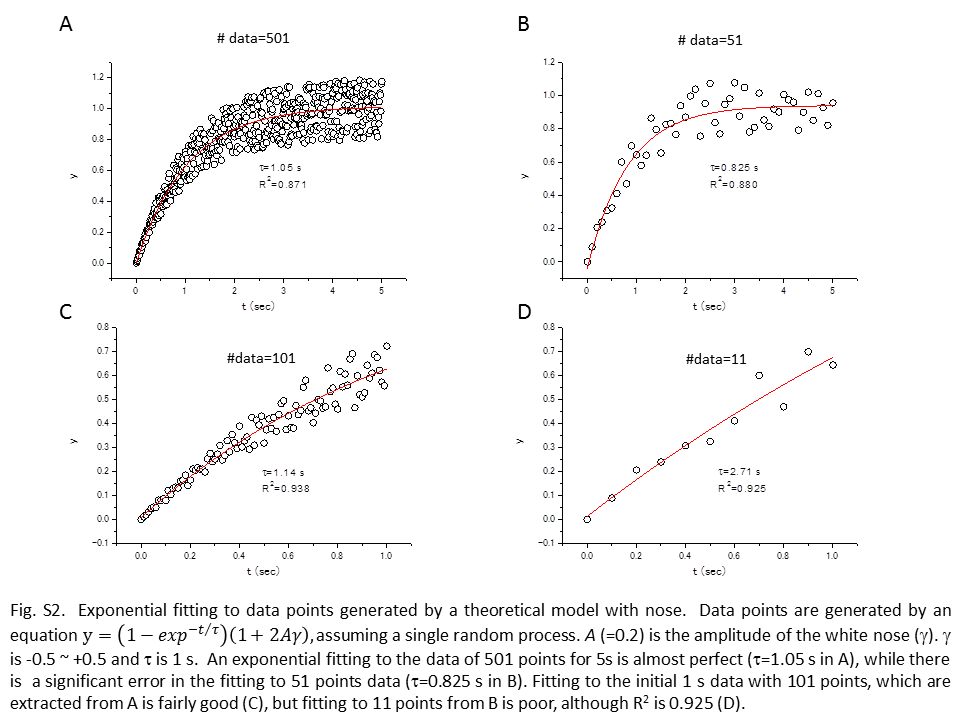

Supplement: S2 Fig — Data points are generated by an equation y = (1-exp -t/τ)(1 + 2Aγ), assuming a single random process. A (= 0.2) is the amplitude of the white nose (γ). γ is-0.5 ~ +0.5 and τ is 1 s. An exponential fitting to the data of 501 points for 5 s is almost perfect (τ = 1.05 s in A), while there is a significant error in the fitting to 51 points data (τ = 0.825 s in B). Fitting to the initial 1 s data with 101 points, which are extracted from A is fairly good (C), but fitting to 11 points from B is poor, although R2 is 0.925 (D). (TIF) [file pone.0116637.s003.tif]
